# Multiple mini interviews: revealing similarities across institutions

**DOI:** 10.1186/s12909-018-1298-8

**Published:** 2018-08-06

**Authors:** Barbara Griffin, Jaime Auton, Robbert Duvivier, Boaz Shulruf, Wendy Hu

**Affiliations:** 1 Dept of Psychology, Macquarie University, Sydney, Australia; 2 University of Newcastle, Newcastle, Australia; 3 Faculty of Medicine, University of NSW, Sydney, Australia; 4 School of Medicine, Western Sydney University, Sydney, Australia

**Keywords:** Multiple mini interviews, Construct validity, Panel interviews

## Abstract

**Background:**

Across the globe multiple mini interviews (MMIs) have rapidly replaced the use of panel interviews in the selection of medical students and other health professionals. MMIs typically demonstrate better reliability and validity than panel interviews but there is limited research on whether these different types of interview process measure the same or different constructs. Our research aims to ascertain if MMIs are multidimensional or unidimensional, and whether MMIs conducted at different institutions assess the same or different constructs to each other or to panel interviews.

**Methods:**

Participants were applicants to medical degrees who were shortlisted for interviews at three different institutions in 2013 (*n* = 165) and 2014 (*n* = 128). Two institutions used a bespoke MMI developed independently from each other and the third used a panel interview. Stations scores and overall (mean) interview scores were examined.

**Results:**

Exploratory principal components analysis and confirmatory factor analysis showed similar results in both years’ data, supporting a unidimensional model. The two overall MMI scores were more strongly correlated to each other (*r* = .56 and .64 in 2013 and 2014 respectively) than either were to the panel interview scores (*r* = .07 and .15 in 2013; .39 and .48 in 2014).

**Conclusions:**

It appears that both MMIs panel interviews tap a single latent construct, but not the same construct. We suggest that the MMI methodology might allow the measurement of an emergent construct such as adaptability.

**Electronic supplementary material:**

The online version of this article (10.1186/s12909-018-1298-8) contains supplementary material, which is available to authorized users.

## Background

Recognising that medical practitioners require more than cognitive or academic ability, universities across the globe have sought to include assessments of non-cognitive qualities, such as empathy and interpersonal skills, as part of their processes for selecting medical students. Panel interviews were widely used to this end. However, even though panel interviews show acceptable psychometric qualities for selection in corporate settings [1], evidence indicates that in the context of high stakes medical student selection they demonstrate low reliability and uncertain predictive validity [2–4]. To overcome these limitations, a team at McMaster University [5] developed the Multiple Mini Interview (MMI), an Objective Structured Clinical Examination (OSCE)-like process whereby candidates progress through a series of multiple, short-lasting stations with typically one interviewer per station who assesses each candidate as they move through one by one. A growing body of research indicates that, compared to panel interview scores, MMI scores typically show better reliability^5^ and predictive validity [6–9]. Moreover, the MMI process is cost effective [10] and is positively evaluated by both interviewees and interviewers [11]. Such evidence helps explain why this new form of interview was rapidly adopted, not only for selection into primary medical degrees, but also into medical specialist training (e.g., General Practice [12]; Obstetrics/Gynaecology, Internal Medicine, and Paediatrics [13]; Ear Nose and Throat [14]; Emergency Medicine [15]) and more recently, by other healthcare professions (e.g., nurses [9, 16]; dentists [17]).

However, it is somewhat surprising that this enthusiastic uptake continues despite scant research on construct validity [4]. In other words, health professionals across the globe are being selected via MMIs when it is still unclear as to what they are actually measuring and whether they assess a different construct/s than the more traditional panel interview.

At a broad level, interviews (both MMIs and the more traditional panel interviews)are thought to be assessing qualities that are non-cognitive in nature [5]. While there is some debate over the use of the term ‘non-cognitive’ [18], the dimensions that institutions report as underpinning interview development (e.g., empathy, ethical values, interpersonal skill) are typically unrelated to cognitive ability or academic performance [19, 20]. There are some exceptions however, with qualities such as “decision-making” not infrequently listed as being the focus of at least one station in an MMI. Beyond this broad domain, the dimensionality of MMIs has received less attention. The current study therefore considers three important questions that have been raised in relation to this aspect of the construct validity of MMIs. First, are they a multidimensional or a unidimensional measure? Second, are MMIs equivalent or is there no relationship between MMIs conducted at different institutions? And third, if MMIs are equivalent, do they measure the same or a different construct to what the traditional panel interview does?

The dimensionality of MMIs has implications for guiding the identification of the specific construct/s being measured, and importantly, for the use of MMI scores. Currently, it would appear that in both research and practice, an overall score (summed across stations) is the most common way of treating an MMI assessment, but this may not be justified if it is in fact, multidimensional [39]. Typically, MMIs are developed by first identifying a set of dimensions that are important to the profession or the institutional context [19], which then become the focus for station/question content. If these are unique or thought to be generally unrelated qualities, then an MMI would appear to assess multiple dimensions. Multi-dimensionality in MMIs has some recent empirical support [21–24], albeit in single institution studies. If multidimensional, one would expect factor analysis of MMI scores to produce more than one conceptually meaningful factor.

The alternative argument to multi-dimensionality is that MMIs actually represent only one overall latent construct. Some have suggested such a construct might be ‘suitability to be a doctor’ or ‘professionalism’ [25]. In this case, stations scores would be indicators of the latent construct. The implication is that MMIs would be similar to a multi-item test, such as a personality test (or scale) of extraversion. Construct validity for a new extraversion scale is demonstrated by its overall positive correlation with established scales – although individual items/questions are different, they are valid samples of the one common underlying factor. Psychometrically, this occurs because aggregation of item scores minimises uncorrelated variance (error) and increases correlated, construct-relevant variance [26]. If such a general dimension exists in MMI data, then we would expect that in addition to factor analytic evidence, the overall scores for MMIs conducted by different institutions to be correlated. Gafni et al. [27] demonstrated such a relationship, but both MMIs in that study were designed by the same team of developers, which may have accounted for the significant correlation.

The second question regarding equivalence has been investigated less frequently, given that the bulk of studies are set in a single institution. While some institutions use the original McMaster MMI, most have developed bespoke versions in terms of station content and the dimensions/qualities they aim to assess. If multidimensional, then MMIs across different institutions are unlikely to be directly comparable, or at least only to the extent of their overlap in measured dimensions [6]. If however, MMIs assess a single or unidimensional construct (either, as discussed below, because of a method factor or because they are tapping a broader latent construct), we would expect MMI scores obtained in different institutions to be related. Indeed, one recent study [28] found a correlation of .47 between MMIs conducted at two different schools.

The third question raises the potential that differences in methodology may result in MMIs and traditional panel interviews measuring a different construct. Regardless of whether panel interviews are designed to assess multiple qualities or one, having the same interviewers rate the candidate on all qualities creates a degree of independence and halo bias that are likely to prevent multiple dimensions being identifiable in the data. However, if MMIs are in fact tapping a general latent construct, such as “potential to be a good doctor/health practitioner” where the specific dimensions are acting as indicators of that construct, they are likely to relate to panel interviews aiming to assess the same construct. A recent study [19] showed this to be the case, but again in a single institution context. Alternatively, a process factor might emerge as a result of the MMI methodology. The MMI requires a candidate to move quite rapidly between stations where they must interact with different interviewers (who likely have different personalities and interpersonal styles), quickly understand and complete different tasks (e.g., scenario-based stations, role-plays, behavioural interviews, film clips, group tasks), and exhibit different behaviours and qualities (e.g., empathy, altruism, teamwork). Overall MMI performance might therefore be an indicator of good adaptability. In contrast, panel interviews require initial adjustment to a group of interviewers but they, and the style of interview, remain constant thereafter so unlikely to assess adaptability as its overall dimension. Jerant et al. [28] provided initial evidence that traditional panel interviews were less strongly related to each other and to MMIs than were MMIs to each other. While we cannot identify the specific factor in the current study, we aim to provide further evidence as to whether or not a similar construct is being measured independent of method.

The current paper answers calls [18, 20] for cross-institution studies to investigate the construct validity of MMIs. Using a dataset of interview scores from three different medical programs in Australia, we examine whether MMIs assess multiple or single constructs and whether these are related to panel interviews; or if MMIs are unrelated to panel interviews or even to each other.

## Method

### Participants and procedure

The participants in this study were applicants to three Australian undergraduate (school leaver) medical degrees in two consecutive years. Although an external body manages final offers, individuals must apply directly to each institution they would like to study at and they can apply to any one or all three institutions (or to any of the other six undergraduate medical degrees in the country). Shortlisting for interview is managed independently and differently by each of the participating institutions, although each uses a combination of the Australian Tertiary Admissions Rank (ATAR; a percentile ranking based on final high school grades) and the Undergraduate Medical and Health Sciences Admissions Test (UMAT; a cognitive ability test with three sections assessing problem solving, understanding people, and abstract reasoning [29]). Relative weightings given to these two measures differ across the three institutions but the separate processes nonetheless result in a subset of applicants who attend interviews at all three universities.

In total, 1092 applicants were interviewed in the first year and 1001 in the second year. Of these, 258 and 251 were interviewed at two institutions in 2013 and 2014 respectively, while 165 and 128 were interviewed by all three institutions. The applicants interviewed three times, of whom 40.6 and 43.8% were female (in 2013 and 2014), form the two primary samples whose data were analysed in this current paper. They are all non-indigenous “domestic” applicants – international applicants and indigenous Australians have a different selection process.

### Measures

The data used for this study are administrative. Nonetheless we obtained ethics approval from each participating institution to conduct the study. The data were merged and deidentified by researchers not employed in any of the three medical schools to ensure complete anonymity.

Two of the universities conduct an MMI and the third runs a panel interview. Interview question development occurs independently at each institution, but some of the qualities targeted for assessment (e.g., motivation, communication skill) are common across interviews. To the best of our knowledge, there was no overlap of interviewers across the three institutions. Interviewers received training designed and provided by each institution, with content and trainers not shared between institutions.

The panel interview (PI) took approximately 40 min with two interviewers who rated each candidate on six different dimensions, all of which were classified as being non-cognitive. One of the MMIs (MMI_1) had nine stations, each lasting for eight minutes and assessing a different dimension. One (decision-making) could be classified as being, at least in part, from the domain of cognitive qualities. The other MMI (MMI_2) had eight by eight minute stations assessing different dimensions with a further cross-station rating of communication. However, three dimensions in MMI_2 were more cognitive in nature. Therefore, when comparing scores we did not include the cognitive dimensions from the two MMIs in order to make the comparison with the PI more equivalent as assessments of the overall ‘non cognitive’ domain. There were two dimensions common to all three interviews: motivation and communication. MMI_1 and MMI_2 had an additional two dimensions in common (one of which was decision-making so not analysed as it is a cognitive skill), MMI_1 and PI had another one in common, and MMI_2 and PI also had an additional one in common. Altogether, 11 different non-cognitive dimensions were included across the three interviews, including ‘motivation’ and ‘communication’ (the two common dimensions), teamwork, ethics, integrity, empathy, etc). Scoring differed across the institutions, and therefore we standardised scores within each institution before conducting the analyses.

### Data analysis approach

To examine the dimensionality of the interview ratings, principal components analyses with varimax rotation were conducted on each institution’s set of non-cognitive station/dimension scores (with separate analyses for the 2013 and 2014 data). For the analysis of the PI, six scores were included, for MMIs 1 and 2 eight and six scores respectively were used.

To examine the relationships between the two MMIs and the PI, we first conducted confirmatory factor analyses (CFAs), with one set on the 2013 data and one set on the 2014 data. Given our aims and the results from the principal components analyses, for each year’s data we compared a one factor model (all dimension/station scores from all institutions loading onto one factor) with a 3-factor model (one overall dimension per institution) and a 2-factor model (the PI dimensions loading onto one factor and MMI_1 and MMI_2 dimensions onto the second factor). Using the cut-off criteria supplied by Hu and Bentler [38], goodness-of-fit was assed using comparative fit index (CFI > .95), Tucker-Lewis index (TLI > .95), standardised root mean square residual (SRMR < .09) and root-mean-square errors of approximation (RMSEA < .06). Competing models were compared using a χ-squared difference test.

Correlation coefficients were then used to assess the relationships between station scores and between overall MMI/PI scores (using the average score). Because the aim of this research is to examine relationships between constructs, we present correlations corrected for unreliability, using the same reliability (*r* = .70) for both the panel interview and the MMIs based on a meta-analysis of employment interviews [30] and the average of the reported reliabilities (*r* = .71) in the recent Best Evidence Medical Education review of MMIs [20].

Statistical significance was set at .05.

## Results

### Dimensionality

The principal components analyses of the 2013 PI data revealed one factor only, which explained 78.09% of the variance. Results for the 2014 PI data were similar, with the one factor explaining 74.81% of the variance. Two factors emerged in both years for MMI1, explaining 42.92 and 44.11% of the variance. The MMI2 data showed two factors in 2013 (42.76% of the variance) but three factors in 2014 (53.10% of variance explained). Although these results suggest a measure of multi-dimensionality in the MMI scores, the results were not consistent across years with different stations loading on different factors in different years and the meaning of each factor was not conceptually clear. The results were unchanged when an oblimin rotation was used.

### Relationship between interviews

#### Confirmatory factor analyses

Results of the CFA analyses are reported in Additional file 1: Appendix. In both years the 2-factor model was the best fit to the data (where all station ratings from both MMIs loaded onto one factor and all ratings from the PI loaded onto the second factor), suggesting that the MMI scores are tapping a different latent construct than the panel interview.

#### Correlations

Table 1 presents the corrected correlations between common qualities across the three universities over the two years, of which 40% were significant in 2013 and 54% in 2014. Of the 122 non-matching pairs of correlations (i.e., between qualities that were conceptually dissimilar) across all interviews, 9.8 and 32.7% were significantly correlated in 2013 and 2014 respectively. However, the majority of correlations for matched pairs were of low effect size, with the average size of the uncorrected correlation between matched dimensions being .098 in 2013 and .152 in 2014. In contrast, the average correlation between the PI’s dimensions in 2013 and 2014 were .737 and .630; on MMI_1 the average correlation between stations in the two years was .189 and .136; and for MMI_2 they were .106 and .123. These results suggest generally greater within-interview than between-interview relationship, even though there appeared to be some between-interview associations when conceptually similar constructs were being compared.

**Table 1:** Corrected correlations between dimension scores (non-cognitive dimensions only)

	2013			2014		
Dimension	PI with MMI_1	PI with MMI_2	MMI_1 with MMI_2	PI with MMI_1	PI with MMI_2	MMI_1 with MMI_2
Motivation	−.10	.15	.29**	.16	.34**	.40**
Communication	.02	.00	.20	.26*	.40**	.03
C	.09			.13		
D	.26*			.28*		
E		.10			.12	
F			.17			.06
Number non-matching correlations	44	33	45	44	33	44
Number (%) significant non-matching correlations	2 (4.5%)	0 (0%)	10 (22%)	20 (45%)	9 (27%)	11 (25%)

*Note*. *p < .05, **p < .001; Dimensions C, D, E, F are unnamed at request of participating institutions

Table 2 reports the corrected correlations between overall scores for both years, where it can be seen that the two MMI scores were more strongly correlated than either were with the panel interview (but less clearly so in the 2014 data). This result supports the CFA analysis showing greater similarity between MMIs than between a total MMI score and a panel interview score.

**Table 2:** Corrected correlations between overall interview scores (non-cognitive dimensions only)

	PI	MMI_1	MMI_2
PI	–	.07	.15
MMI_1	.48**	–	.56**
MMI_2	.39**	.64**	–

*Note.* **p < .001

Values above the diagonal = 2013 data, below the diagonal = 2014

## Discussion

This multi-institutional study addressed issues related to the investigation of the construct validity of MMIs, using data from a group of medical school applicants who were interviewed for entry into three independent medical degrees. In particular, we sought to provide information on the dimensionality of interview ratings, to ascertain if medical school applicants performed similarly on two different MMIs conducted at two different institution, and to assess if the latent construct/s being assessed by MMIs and traditional panel interviews were similar.

We compared scores from two MMIs and a panel interview, with the results showing little support for the idea that MMIs (or the panel interview) assess multiple unique dimensions that are conceptually clear. Rather, our analyses suggest that panel interviews are more likely to measure a general overall/single dimension and that whilst MMI data formed more than one factor, these were not conceptually clear or consistent, and did not correspond to the number of qualities the MMIs were originally designed to assess. Moreover, when subject to a CFA, a single factor solution fit well. Furthermore, the confirmatory factor analyses suggested that the two different MMIs appear to be tapping the same latent construct. Correlations revealed that this underlying MMI factor seemed to have little relationship to scores on the panel interview – results that support the single institution study by Bibler et al. [31] and a multi-institutional study by Jerant et al. [28].

To assist in interpreting these results, we draw on the large body of literature on assessment centers (ACs). ACs, which are widely used for selection and development in the corporate world [32], are similar to MMIs in that they consist of several stations (called ‘exercises’) designed to assess several dimensions. Even though exercises can be longer lasting than MMI stations, the process is conceptually analogous. After much debate regarding ACs’ ability to measure multiple dimensions across exercises, Kuncel and Sackett [26] provided conceptual and mathematical support for a unidimensional view by showing that, as the number of exercises/stations increases, a general dimension factor emerges to dominate the variance in the data (i.e., dimension-specific variance and error variance become relatively smaller and less consequential). Dimension-specific variance refers to that explaining individual criteria such as teamwork, motivation, communication, interpersonal skills. We refer readers to the AC literature in the hope that MMI researchers will not need to repeat the same debate.

It was beyond the scope of our study to name the general dimension identified in the MMI data. However, considering that it appears to differ from that captured by panel interviews, the MMI might assess a quality that emerges as a result of the particular demands within the process, which are different to the traditional panel interview process. We suggested one possibility might be adaptability. Alternatively, Kuncel and Sackett^26^ suggest the general dimension that emerges in ACs (and therefore likely in MMIs) could be the newly-identified construct ‘ability to identify criteria’ (ATIC) [33, 34]. ATIC is defined as the “ability to correctly perceive performance criteria when participating in an evaluative situation”. [35] (p129) Those candidates with high ATIC are better at picking up environmental cues and therefore more quickly understand what behaviour is required of them in a given context. MMIs are context specific [6] with rapid changes in contextual demands. Griffin^34^ demonstrated that high ATIC predicted better MMI scores in a group of medical school applicants. While ATIC may also enable performance in a panel interview [35] the stable context should reduce its usefulness and the likelihood that it is captured as the general dimension. These ideas are of course dependent on the stations within an MMI having different demands as is the case with AC designs. Some MMIs have been developed with every station requires the candidate to respond to a scenario-based hypothetical dilemma. In such cases, the underlying latent dimension has been described as ‘entry-level reasoning skills in professionalism.’ [36]

The results of this study showed that even when interviews are designed to assess the same sub dimensions (e.g., communication) there is little correlation between scores at different institutions. It is quite possible that the definition and interpretation of what a sub dimension means and how it is best assessed differs substantially between institutions and as a result of a local MMI development process. These differences might explain why, for example, ‘motivation’ measured at one school in a traditional panel interview did not relate to the MMI measures of motivation. Even though the results of this study do not support a multi-dimensional view of MMIs (or panel interviews) where dimensions correspond to the number of qualities the interview was designed to assess, more evidence is required before we would advocate abandoning the practice of identifying a set of important dimensions/criteria to guide station/question development. In particular, this can focus ideas and evaluation, but further work on clarifying definitions of important qualities could not only assist in uniformity but guide interview development teams. However, we suggest that the results of the current study provide support for the aggregation of dimension or station scores into an overall score, and that this overall score be used for ranking applicants or as the basis of providing them with feedback. Psychometrically, aggregation will reduce random error variance and the unwanted specific variance associated with any one station [26].

A practical implication of the finding that the two overall MMI scores correlated at moderate to high effect size, is that university admission committees could reliably reduce time and costs by ‘sharing’ overall MMI scores for applicants who apply to several institutions. For example, the two universities in this study who conducted an MMI actually interviewed 277 of the same individuals. They could conceivably split this shared pool to interview only half that number each. However, conducting MMIs often has a secondary aim, such as the introduction of applicants to a medical school’s learning environment, staff and ethos, which may influence an applicants’ actual choice of medical school if they are offered a place in more than one institution.

The findings of high correlations between the different dimensions rated in the panel interview and high loadings on the one factor in the CFA and principal components analysis highlight the difficulty panel interviewers have in distinguishing between dimensions [37].

### Limitations

Whilst this study’s use of multi-institutional is a strength, the large pool of applicants was reduced to relatively low numbers who had interviews at all three institutions (*n* < 170 in both samples). It is also important to note that both MMIs assessed a single dimension at each station (except for communication at MMI_2). This means, unlike ACs and MMIs that assess all dimensions of interest at each station, in the current study station and dimension scores were confounded. Nevertheless, given Kuncel and Sackett’s^26^ evidence, we would expect the same results, perhaps even stronger, from the alternative design. Further, even though the findings were consistent across two years with unique participants, there was only one PI and two MMIs so the factor structure will need to be confirmed in other data.

## Conclusion

This study analysed multi-institution data to assess the relationship between two MMIs and a panel interview, each of which was designed to assess multiple criteria. The results provide more support for interviews being measures of one underlying dimension rather than a set of several conceptually clear dimensions. Furthermore, MMIs designed and run at two independent institution showed a degree of similarity (and difference from the panel interview), suggesting that they are likely to assess the same overall dimension.

## Additional file

Additional file 1: Appendix 1 Fit indices for competing models. (DOCX 39 kb)

## Abbreviations

AC: Assessment Center
ATIC: Ability to identify criteria
CFA: Confirmatory factor analysis
CFI: Comparative Fit Index
MMI: Multiple Mini Interview
OSCE: Objective Structured Clinical Examination
PI: Panel interview
RMSEA: Root mean square errors of approximation
SRMR: Standardised root mean square residual
TLI: Tucker-Lewis index
UMAT: Undergraduate Medical and Health Sciences Admissions Test
