# Supplementary material for: Multiple mini interviews: revealing similarities across institutions
Source: BMC Med Educ. 2018 Aug 6;18:190. doi: 10.1186/s12909-018-1298-8 (PMC6080397; doi:10.1186/s12909-018-1298-8)
Supplement: Supplementary file 1 — Appendix 1 Fit indices for competing models. (DOCX 39 kb) [file 12909_2018_1298_MOESM1_ESM.docx]

**Additional file**

**Multiple Mini Interviews: Revealing similarities across institutions**

**Additional file1: Appendix 1** Fit Indices of Competing Factor Models for 2013 and 2014

| Model | *χ^2^* | *df* | CFI | TLI | RMSEA | SRMR | Model Comparison | Δ*df* | Δ*χ^2^* |
| --- | --- | --- | --- | --- | --- | --- | --- | --- | --- |
| 2013 | | | | | | | | | |
| M_1 Factor_ | 303.229** | 170 | .87 | .85 | .07 | .09 |  |  |  |
| M_2 Factor_ | 190.985 | 169 | .98 | .98 | .03 | .06 | M_1 Factor_- M_2 Factor_ | 1 | 112.244* |
| M_3 Factor_ | 185.173 | 167 | .98 | .98 | .03 | .06 | M_3 Factor_- M_1 Factor_ | 3 | 118.056* |
|  | | | | | | | M_3 Factor-_ M_2 Factor_ | 2 | 5.812 |
| 2014 | | | | | | | | | |
| M_1 Factor_ | 253.763** | 152 | .78 | .76 | .07 | .09 |  |  |  |
| M_2 Factor_ | 192.541* | 151 | .91 | .90 | .05 | .08 | M_1 Factor_- M_2 Factor_ | 1 | 61.222* |
| M_3 Factor_ | 188.970* | 149 | .92 | .90 | .05 | .08 | M_3 Factor_- M_1 Factor_ | 3 | 64.793* |
|  | | | | | | | M_3 Factor-_ M_2 Factor_ | 2 | 3.571 |

*Note*. CFI=comparative fit index; TLI=Tucker-Lewis index; RMSEA=root mean square error of approximation; SRMR=standardized root mean square residual.

**p*<.05; ***p*<.01.
